# Supplementary material for: Rhodoalgimonas zhirmunskyi gen. nov., sp. nov., a Marine Alphaproteobacterium Isolated from the Pacific Red Alga Ahnfeltia tobuchiensis: Phenotypic Characterization and Pan-Genome Analysis
Source: Microorganisms. 2023 Sep 30;11(10):2463. doi: 10.3390/microorganisms11102463 (PMC10608839; doi:10.3390/microorganisms11102463)
Supplement: Supplementary file 1 [file microorganisms-11-02463-s001.zip › microorganisms-2590737-supplementary.pdf]

Rhodoalgimonas zhirmunskyi gen. nov., sp. nov., a marine alphaproteobacterium isolated from the Pacific red alga *Ahnfeltia tobuchiensis*: phenotypic characterization and pan-genome analysis

Olga Nedashkovskaya [1*], Nadezhda Otstavnykh [1], Larissa Balabanova [1], Evgenia Bystritskaya [1], Song-Gun Kim [2], Natalia Zhukova [3], Liudmila Tekutyeva [4,5], and Marina Isaeva [1,*]

1 G.B. Elyakov Pacific Institute of Bioorganic Chemistry, Far Eastern Branch, Russian Academy of Sciences, Prospect 100 Let Vladivostoku, 159, Vladivostok 690022, Russia; olganedashkovska@piboc.dvo.ru (O.N.); lbalabanova1@gmail.com (L.B.); chernysheva.nadezhda@gmail.com (N.O.); belyana@gmail.com; (V.K.); issaeva@piboc.dvo.ru (M.I.)

2 Korean Collection for Type Cultures, Biological Resource Center, Korea Research Institute of Bioscience and Biotechnology, 181 Ipsin-gil, Jeongeup-si, Jeollabuk-do 56212, Republic of Korea; sgkim@kribb.re.kr (S.K.)

3 A.V. Zhirmunsky National Scientific Center of Marine Biology, Far Eastern Branch, Russian Academy of Sciences, Palchevskogo Street 17, Vladivostok 690041, Russia; nzhukova35@list.ru (N.Z.)

4 Innovative Technology Center, Far Eastern Federal University, 8 Suhanova St., Vladivostok 690950, Russia; tekuteva.la@dvfu.ru (L.T.)

5 ARNIKA, Territory of PDA Nadezhdinskaya, Centralnaya St. 42, Volno-Nadezhdinskoye, Primorsky Krai, 692481 Vladivostok, Russia; tekuteva.la@dvfu.ru (L.T.)

*Correspondence: olganedashkovska@piboc.dvo.ru (O.N.); issaeva@gmail.com (M.I.); Tel.: +7-423-231-1168 (O.N.)

Supplementary

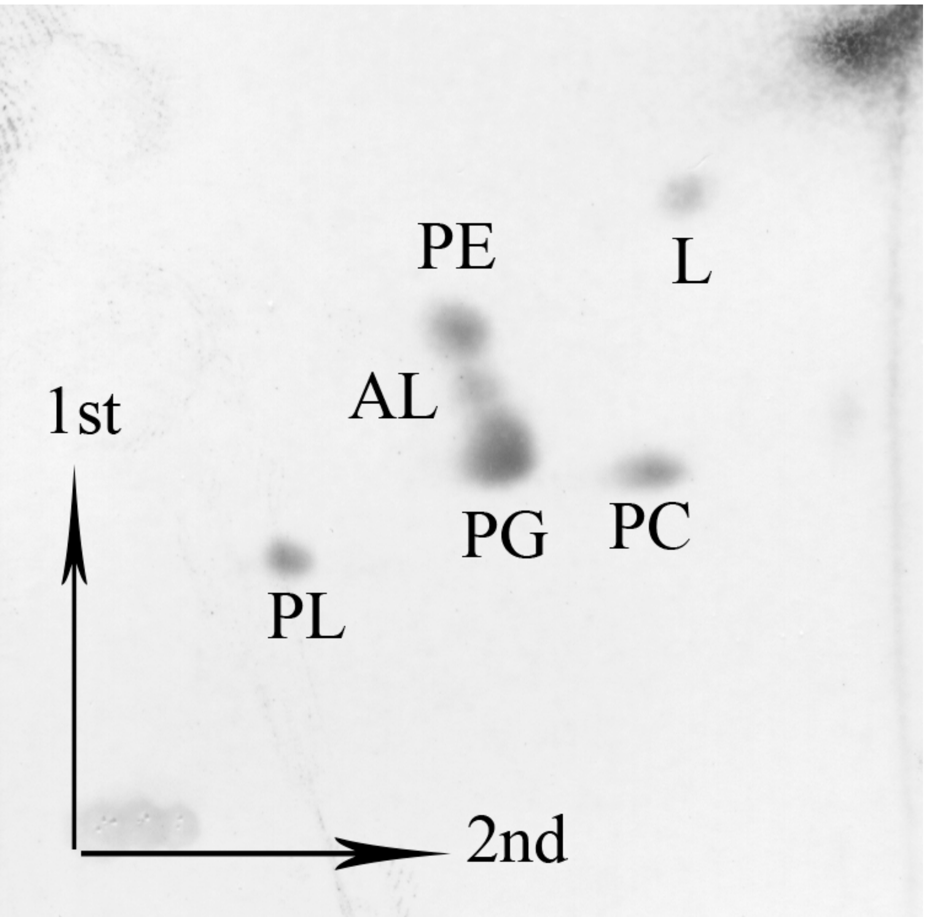

Supplementary Figure S1. Two-dimensional TLC of the total polar lipids of strain 10Alg 79^{T}. First dimension, chloroform/methanol/water (65: 25: 4, v/v/v); second dimension, chloroform/methanol/acetic acid/water (80: 12: 15: 4, v/v/v/v). PE – phosphatidylethanolamine, PG – phosphatidylglycerol, PC – phosphatidylcholine, AL – an unidentified aminolipid, PL, an unidentified phospholipid, and L, an unidentified lipid.
